# Supplementary material for: Comparative assessment of the cost-effectiveness of Tuberculosis (TB) active case-finding interventions: A systematic analysis of TB REACH wave 5 projects
Source: PLoS One. 2022 Sep 26;17(9):e0270816. doi: 10.1371/journal.pone.0270816 (PMC9512197; doi:10.1371/journal.pone.0270816)
Supplement: S1 File — (DOCX) [file pone.0270816.s001.docx]

**Supporting Information**

**Table S1. Treatment initiation and cost-effectiveness of TB REACH Wave 5 projects by project type.**

| **#** | **Project Code** | **Region^a^** | **Setting (Target Population)^b^** | **Apportioned Costs** | **Number of Patients Started on Treatment** | **Cost per Case Initiated Treatment^c^** |
| --- | --- | --- | --- | --- | --- | --- |
| **Case-finding & Linkage-to-Care** | | | | | | |
| **S4** | **ASHAK** | SEAR | Rural (Indigenous populations) | $321,924 | 2,626 | $123 |
| **A5** | **LSTME** | AFR | Rural | $192,259 | 599 | $321 |
| **S9** | **RUMAH** | SEAR | Urban (Children) | $188,183 | 520 | $362 |
| **A3** | **CIDRZ** | AFR | Urban | $498,932 | 1027 | $486 |
| **A7** | **GLRAN** | AFR | Urban (Mothers, HIV patients, Outpatients) | $157,890 | 319 | $495 |
| **S7** | **OPASH** | SEAR | Rural | $321,924 | 648 | $497 |
| **W3** | **VNTPV** | WPR | Urban | $726,634 | 1,139 | $638 |
| **W4** | **FITVT** | WPR | Urban | $130,135 | 165 | $789 |
| **A12** | **GLOHI** | AFR | Rural | $243,985 | 23 | $10,608 |
| **Average cost ratio** | | | | | | $394 |
| **Case-finding, Linkage-to-Care & Patient Support** | | | | | | |
| **W1** | **CATAC** | WPR | Rural (Elderly population) | $414,704 | 2,669 | $155 |
| **S5** | **INNOV** | SEAR | Rural | $308,777 | 1,641 | $188 |
| **W2** | **KHANA** | WPR | Urban | $316,420 | 1,614 | $196 |
| **A1** | **HEAAI** | AFR | Urban | $491,441 | 1,507 | $326 |
| **A4** | **SHDEP** | AFR | Urban (General population; Children, Female Sex Workers, Small-Scale Miners, MSM) | $295,736 | 863 | $343 |
| **E3** | **BRICF** | EMR | Urban (Transgender People, Male Sex Workers) | $237,799 | 614 | $387 |
| **S6** | **BNMTN** | SEAR | Rural (High Risk populations) | $481,011 | 1,061 | $453 |
| **A11** | **NAANK** | AFR | Rural | $51,528 | 24 | $2,147 |
| **P1** | **ASOCI** | PAR | Urban | $309,155 | 72 | $4,294 |
| **Average cost ratio** | | | | | | $289 |
| **Average cost ratio (All Projects)** | | | | | | $332 |

a. Region is grouped by the WHO definition: African Region (AFR), Region of the Americas (PAR), South-East Asia Region (SEAR), European Region (EUR), Eastern Mediterranean Region (EMR), and Western Pacific Region (WPR).

b. Projects are categorized into urban or rural setting based on the primary implementation environments. Targeted populations are specified in TB REACH narrative reports.

c. Cost per treatment initiation is calculated as total project costs divided by the number of patients initiated on treatment.

**Table S2. Treatment linkage outcome and cost-effectiveness ratio of TB REACH Wave 5 projects by project type.**

| **#** | **Project Code** | **Region^a^** | **Setting (Target Population)^b^** | **Apportioned Costs** | **Number of Patients Started on Treatment** | **Cost per Care Linkage^c^** |
| --- | --- | --- | --- | --- | --- | --- |
| **Case-finding & Linkage-to-Care** | | | | | | |
| **W3** | **VNTPV** | WPR | Urban | $10,860 | 1,139 | $10 |
| **S4** | **ASHAK** | SEAR | Rural (Indigenous populations) | $52,254 | 2,626 | $20 |
| **W4** | **FITVT** | WPR | Urban | $3,796 | 165 | $23 |
| **A7** | **GLRAN** | AFR | Urban (Mothers, HIV patients, Outpatients) | $11,649 | 319 | $37 |
| **A5** | **LSTME** | AFR | Rural | $24,740 | 599 | $41 |
| **S9** | **RUMAH** | SEAR | Urban (Children) | $22,538 | 520 | $43 |
| **A3** | **CIDRZ** | AFR | Urban | $66,421 | 1,027 | $65 |
| **S7** | **OPASH** | SEAR | Rural | $53,216 | 648 | $82 |
| **A12** | **GLOHI** | AFR | Rural | $15,988 | 23 | $695 |
| **Average cost ratio** | | | | | | $37 |
| **Case-finding, Linkage-to-Care & Patient Support** | | | | | | |
| **W1** | **CATAC** | WPR | Rural (Elderly population) | $20,780 | 2,669 | $8 |
| **S6** | **BNMTN** | SEAR | Rural (High Risk populations) | $17,754 | 1,061 | $17 |
| **A4** | **SHDEP** | AFR | Urban (General population; Children, Female Sex Workers, Small-Scale Miners, MSM) | $16,654 | 863 | $19 |
| **S5** | **INNOV** | SEAR | Rural | $32,209 | 1,641 | $20 |
| **E3** | **BRICF** | EMR | Urban (Transgender People, Male Sex Workers) | $15,569 | 614 | $25 |
| **W2** | **KHANA** | WPR | Urban | $70,801 | 1,614 | $44 |
| **A1** | **HEAAI** | AFR | Urban | $78,947 | 1,507 | $52 |
| **P1** | **ASOCI** | PAR | Urban | $5,236 | 72 | $73 |
| **A11** | **NAANK** | AFR | Rural | $2,193 | 24 | $91 |
| **Average cost ratio** | | | | | | $26 |
| **Average cost ratio (All Projects)** | | | | | | $30 |

a. Region is grouped by the WHO definition: African Region (AFR), Region of the Americas (PAR), South-East Asia Region (SEAR), European Region (EUR), Eastern Mediterranean Region (EMR), and Western Pacific Region (WPR).

b. Projects are categorized into urban or rural setting based on the primary implementation environments. Targeted population is specified when being emphasized in the TB REACH narrative reports.

c. Cost per care linkage is calculated as respective linkage-to-care costs divided by a number of patients started on treatment.

**Table S3. Treatment completion and cost-effectiveness ratio of TB REACH Wave 5 projects (with treatment support costs).**

| **#** | **Project Code** | **Region^a^** | **Setting (Target Population)^b^** | **Apportioned Costs** | **Number of Patients Completed Treatment** | **Cost per Case Completed Treatment^c^** |
| --- | --- | --- | --- | --- | --- | --- |
| **Case-finding, Linkage-to-Care & Patient Support** | | | | | | |
| **W1** | **CATAC** | WPR | Rural (Elderly population) | $20,780 | 2,634 | $8 |
| **S6** | **BNMTN** | SEAR | Rural (High Risk populations) | $17,754 | 631 | $28 |
| **E3** | **BRICF** | EMR | Urban (Transgender People, Male Sex Workers) | $15,569 | 552 | $28 |
| **S5** | **INNOV** | SEAR | Rural | $32,209 | 1,099 | $29 |
| **W2** | **KHANA** | WPR | Urban | $70,801 | 839 | $84 |
| **P1** | **ASOCI** | PAR | Urban | $5,236 | 57 | $92 |
| **A4** | **SHDEP** | AFR | Urban (General population; Children, Female Sex Workers, Small-Scale Miners, MSM) | $16,654 | 122 | $137 |
| **A11** | **NAANK** | AFR | Rural | $2,193 | 15 | $146 |
| **A1** | **HEAAI** | AFR | Urban | $78,947 | 494 | $160 |
| **Average cost ratio** | | | | | | $40 |

a. Region is grouped by the WHO definition: African Region (AFR), Region of the Americas (PAR), South-East Asia Region (SEAR), European Region (EUR), Eastern Mediterranean Region (EMR), and Western Pacific Region (WPR).

b. Projects are categorized into urban or rural setting based on the primary implementation environments. Targeted population is specified when being emphasized in the TB REACH narrative reports.

c. Cost per treatment completed is calculated as respective treatment support costs divided by the estimated number of patients completing treatment.

**Table S4. Treatment completion and cost-effectiveness ratio of TB REACH Wave 5 projects (with total costs).**

| **#** | **Project Code** | **Region^a^** | **Setting (Target Population)^b^** | **Apportioned Costs** | **Number of Patients Completed Treatment** | **Cost per Case Completed Treatment^c^** |
| --- | --- | --- | --- | --- | --- | --- |
| **Case-finding, Linkage-to-Care & Patient Support** | | | | | | |
| **W1** | **CATAC** | WPR | Rural (Elderly population) | $414,704 | 2,634 | $157 |
| **S5** | **INNOV** | SEAR | Rural | $308,777 | 1,099 | $281 |
| **W2** | **KHANA** | WPR | Urban | $316,420 | 839 | $377 |
| **E3** | **BRICF** | EMR | Urban (Transgender People, Male Sex Workers) | $237,799 | 552 | $431 |
| **S6** | **BNMTN** | SEAR | Rural (High Risk populations) | $481,011 | 631 | $762 |
| **A1** | **HEAAI** | AFR | Urban | $491,441 | 494 | $995 |
| **A4** | **SHDEP** | AFR | Urban (General population; Children, Female Sex Workers, Small-Scale Miners, MSM) | $295,736 | 122 | $2,424 |
| **A11** | **NAANK** | AFR | Rural | $51,528 | 15 | $3,435 |
| **P1** | **ASOCI** | PAR | Urban | $309,155 | 57 | $5,424 |
| **Average cost ratio** | | | | | | $451 |

a. Region is grouped by the WHO definition: African Region (AFR), Region of the Americas (PAR), South-East Asia Region (SEAR), European Region (EUR), Eastern Mediterranean Region (EMR), and Western Pacific Region (WPR).

b. Projects are categorized into urban or rural settings based on the primary implementation environment. Target populations are specified according to TB REACH narrative reports.

c. Cost per treatment completed is calculated as total project costs divided by the number of patients completing treatment.

**Table S5. Case-finding and cost-effectiveness of TB REACH Wave 5 projects by subgroup.**

| **#** | **Project Code** | **Region^a^** | **Setting (Target Population)^b^** | **Apportioned Costs** | **Number of Patients Diagnosed** | **Cost per Case Diagnosed^c^** |
| --- | --- | --- | --- | --- | --- | --- |
| **Technology** | | | | | | |
| **S3** | **TBALI** | SEAR | Urban | $170,735 | 5,765 | $30 |
| **S2** | **REACH** | SEAR | Urban | $934,125 | 8,675 | $108 |
| **W1** | **CATAC** | WPR | Rural (Elderly population) | $393,924 | 2,801 | $141 |
| **A1** | **HEAAI** | AFR | Urban | $412,494 | 1,516 | $272 |
| **S9** | **RUMAH** | SEAR | Urban (Children) | $165,645 | 532 | $311 |
| **A3** | **CIDRZ** | AFR | Urban | $432,511 | 1030 | $420 |
| **A7** | **GLRAN** | AFR | Urban (Mothers, HIV patients, Outpatients) | $146,241 | 334 | $438 |
| **A9** | **FUNDA** | AFR | Urban | $306,335 | 99 | $3,094 |
| **A12** | **GLOHI** | AFR | Rural | $227,997 | 23 | $9,913 |
| **A10** | **IRDSA** | AFR | Urban (Children, Pregnancy) | $325,415 | 31 | $10,497 |
| **Average cost ratio** | | | | | | $169 |
| **PPM (private sector involvement)** | | | | | | |
| **S3** | **TBALI** | SEAR | Urban | $170,735 | 5,765 | $30 |
| **S1** | **ICDDR** | SEAR | Urban | $783,292 | 17,100 | $46 |
| **S2** | **REACH** | SEAR | Urban | $934,125 | 8,675 | $108 |
| **A8** | **LSTMN** | AFR | Urban | $170,594 | 247 | $691 |
| **W4** | **FITVT** | WPR | Urban | $126,339 | 171 | $739 |
| **Average cost ratio** | | | | | | $68 |
| **Hard-to-reach populations (villages, camps, isolated regions)** | | | | | | |
| **S4** | **ASHAK** | SEAR | Rural (Indigenous populations) | $269,670 | 2,626 | $103 |
| **W1** | **CATAC** | WPR | Rural (Elderly population) | $393,924 | 2,801 | $141 |
| **W2** | **KHANA** | WPR | Urban | $245,619 | 1,620 | $152 |
| **E1** | **MERCY** | EMR | Urban | $269,388 | 1,165 | $231 |
| **A2** | **GOMSA** | AFR | Rural (Internally Displaced Persons) | $335,312 | 1,423 | $236 |
| **S9** | **RUMAH** | SEAR | Urban (Children) | $165,645 | 532 | $311 |
| **A12** | **GLOHI** | AFR | Rural | $227,997 | 23 | $9,913 |
| **Average cost ratio** | | | | | | $187 |
| **Pregnant women/pediatric TB cases** | | | | | | |
| **S1** | **ICDDR** | SEAR | Urban | $783,292 | 17,100 | $46 |
| **W2** | **KHANA** | WPR | Urban | $245,619 | 1,620 | $152 |
| **A2** | **GOMSA** | AFR | Rural (Internally Displaced Persons) | $335,312 | 1,423 | $236 |
| **A1** | **HEAAI** | AFR | Urban | $412,494 | 1,516 | $272 |
| **A4** | **SHDEP** | AFR | Urban (General population; Children, Female Sex Workers, Small-Scale Miners, MSM) | $279,082 | 922 | $303 |
| **S9** | **RUMAH** | SEAR | Urban (Children) | $165,645 | 532 | $311 |
| **A3** | **CIDRZ** | AFR | Urban | $432,511 | 1030 | $420 |
| **E2** | **ACREO** | EMR | Urban (Women) | $287,080 | 626 | $459 |
| **W3** | **VNTPV** | WPR | Urban | $715,774 | 1,400 | $511 |
| **S8** | **MAPIN** | SEAR | Rural | $341,921 | 581 | $589 |
| **A6** | **CHEAS** | AFR | Urban (Children) | $852,498 | 440 | $1,937 |
| **A11** | **NAANK** | AFR | Rural | $49,335 | 24 | $2,056 |
| **A10** | **IRDSA** | AFR | Urban (Children, Pregnancy) | $325,415 | 31 | $10,497 |
| **Average cost ratio** | | | | | | $192 |
| **Door-door screening** | | | | | | |
| **S5** | **INNOV** | SEAR | Rural | $276,568 | 1,730 | $160 |
| **A4** | **SHDEP** | AFR | Urban (General population; Children, Female Sex Workers, Small-Scale Miners, MSM) | $279,082 | 922 | $303 |
| **A3** | **CIDRZ** | AFR | Urban | $432,511 | 1030 | $420 |
| **W3** | **VNTPV** | WPR | Urban | $715,774 | 1,400 | $511 |
| **S8** | **MAPIN** | SEAR | Rural | $341,921 | 581 | $589 |
| **Average cost ratio** | | | | | | $361 |
| **Average cost ratio (All Projects)** | | | | | | $184 |

a. Region is grouped by the WHO definition: African Region (AFR), Region of the Americas (PAR), South-East Asia Region (SEAR), European Region (EUR), Eastern Mediterranean Region (EMR), and Western Pacific Region (WPR).

b. Projects are categorized into urban or rural settings based on the primary implementation environment. Targeted population is specified according to TB REACH narrative reports.

c. Cost per case diagnosed is calculated as respective case-finding costs divided by the number of patients diagnosed.

**Table S6. Treatment initiation and cost-effectiveness of TB REACH Wave 5 projects by subgroup.**

| **#** | **Project Code** | **Region^a^** | **Setting (Target Population)^b^** | **Apportioned Costs** | **Number of Patients Started on Treatment** | **Cost per Case Initiated Treatment^c^** |
| --- | --- | --- | --- | --- | --- | --- |
| **Technology** | | | | | | |
| **W1** | **CATAC** | WPR | Rural (Elderly population) | $414,704 | 2,669 | $155 |
| **A1** | **HEAAI** | AFR | Urban | $491,441 | 1,507 | $326 |
| **S9** | **RUMAH** | SEAR | Urban (Children) | $188,183 | 520 | $362 |
| **A3** | **CIDRZ** | AFR | Urban | $498,932 | 1027 | $486 |
| **A7** | **GLRAN** | AFR | Urban (Mothers, HIV patients, Outpatients) | $157,890 | 319 | $495 |
| **A12** | **GLOHI** | AFR | Rural | $243,985 | 23 | $10,608 |
| **Average cost ratio** | | | | | | $329 |
| **PPM (private sector involvement)** | | | | | | |
| **W4** | **FITVT** | WPR | Urban | $130,135 | 165 | $789 |
| **Average cost ratio** | | | | | | $789 |
| **Hard-to-reach populations (villages, camps, isolated regions)** | | | | | | |
| **S4** | **ASHAK** | SEAR | Rural (Indigenous populations) | $321,924 | 2,626 | $123 |
| **W1** | **CATAC** | WPR | Rural (Elderly population) | $414,704 | 2,669 | $155 |
| **W2** | **KHANA** | WPR | Urban | $316,420 | 1,614 | $196 |
| **S9** | **RUMAH** | SEAR | Urban (Children) | $188,183 | 520 | $362 |
| **A12** | **GLOHI** | AFR | Rural | $243,985 | 23 | $10,608 |
| **Average cost ratio** | | | | | | $199 |
| **Pregnant women/pediatric TB cases** | | | | | | |
| **W2** | **KHANA** | WPR | Urban | $316,420 | 1,614 | $196 |
| **A1** | **HEAAI** | AFR | Urban | $491,441 | 1,507 | $326 |
| **A4** | **SHDEP** | AFR | Urban (General population; Children, Female Sex Workers, Small-Scale Miners, MSM) | $295,736 | 863 | $343 |
| **S9** | **RUMAH** | SEAR | Urban (Children) | $188,183 | 520 | $362 |
| **A3** | **CIDRZ** | AFR | Urban | $498,932 | 1027 | $486 |
| **W3** | **VNTPV** | WPR | Urban | $726,634 | 1,139 | $638 |
| **A11** | **NAANK** | AFR | Rural | $51,528 | 24 | $2,147 |
| **Average cost ratio** | | | | | | $384 |
| **Door-door screening** | | | | | | |
| **S5** | **INNOV** | SEAR | Rural | $308,777 | 1,641 | $188 |
| **A4** | **SHDEP** | AFR | Urban (General population; Children, Female Sex Workers, Small-Scale Miners, MSM) | $295,736 | 863 | $343 |
| **A3** | **CIDRZ** | AFR | Urban | $498,932 | 1027 | $486 |
| **W3** | **VNTPV** | WPR | Urban | $726,634 | 1,139 | $638 |
| **Average cost ratio** | | | | | | $392 |
| **Average cost ratio (All Projects)** | | | | | | $332 |

a. Region is grouped by the WHO definition: African Region (AFR), Region of the Americas (PAR), South-East Asia Region (SEAR), European Region (EUR), Eastern Mediterranean Region (EMR), and Western Pacific Region (WPR).

b. Projects are categorized into urban or rural setting based on the primary implementation environments. Targeted population is specified when being emphasized in the TB REACH narrative reports.

c. Cost per treatment initiation is calculated as total project costs divided by a number of patients initiated on treatment.

**Table S7. Treatment completion and cost-effectiveness of TB REACH Wave 5 projects by subgroup (treatment support costs).**

| **#** | **Project Code** | **Region^a^** | **Setting (Target Population)^b^** | **Apportioned Costs** | **Number of Patients Completed Treatment** | **Cost per Case Completed Treatment^c^** |
| --- | --- | --- | --- | --- | --- | --- |
| **Technology** | | | | | | |
| **W1** | **CATAC** | WPR | Rural (Elderly population) | $20,780 | 2,634 | $8 |
| **A1** | **HEAAI** | AFR | Urban | $78,947 | 494 | $160 |
| **Average cost ratio** | | | | | | $32 |
| **Hard-to-reach populations (villages, camps, isolated regions)** | | | | | | |
| **W1** | **CATAC** | WPR | Rural (Elderly population) | $20,780 | 2,634 | $8 |
| **W2** | **KHANA** | WPR | Urban | $70,801 | 839 | $84 |
| **Average cost ratio** | | | | | | $26 |
| **Pregnant women/pediatric TB cases** | | | | | | |
| **W2** | **KHANA** | WPR | Urban | $70,801 | 839 | $84 |
| **A4** | **SHDEP** | AFR | Urban (General population; Children, Female Sex Workers, Small-Scale Miners, MSM) | $16,654 | 122 | $137 |
| **A11** | **NAANK** | AFR | Rural | $2,193 | 15 | $146 |
| **A1** | **HEAAI** | AFR | Urban | $78,947 | 494 | $160 |
| **Average cost ratio** | | | | | | $115 |
| **Door-door screening** | | | | | | |
| **S5** | **INNOV** | SEAR | Rural | $32,209 | 1,099 | $29 |
| **A4** | **SHDEP** | AFR | Urban (General population; Children, Female Sex Workers, Small-Scale Miners, MSM) | $16,654 | 122 | $137 |
| **Average cost ratio** | | | | | | $40 |
| **Average cost ratio (All Projects)** | | | | | | $40 |

a. Region is grouped by the WHO definition: African Region (AFR), Region of the Americas (PAR), South-East Asia Region (SEAR), European Region (EUR), Eastern Mediterranean Region (EMR), and Western Pacific Region (WPR).

b. Projects are categorized into urban or rural setting based on the primary implementation environments. Targeted population is specified when being emphasized in the TB REACH narrative reports.

c. Cost per treatment completed is calculated as respective treatment support costs divided by a number of patients completed treatment.

**Table S8. Treatment completion and cost-effectiveness of TB REACH Wave 5 projects by subgroup (total costs).**

| **#** | **Project Code** | **Region^a^** | **Setting (Target Population)^b^** | **Apportioned Costs** | **Number of Patients Completed Treatment** | **Cost per Case Completed Treatment^c^** |
| --- | --- | --- | --- | --- | --- | --- |
| **Technology** | | | | | | |
| **W1** | **CATAC** | WPR | Rural (Elderly population) | $414,704 | 2,634 | $157 |
| **A1** | **HEAAI** | AFR | Urban | $491,441 | 494 | $995 |
| **Average cost ratio** | | | | | | $290 |
| **Hard-to-reach populations (villages, camps, isolated regions)** | | | | | | |
| **W1** | **CATAC** | WPR | Rural (Elderly population) | $414,704 | 2,634 | $157 |
| **W2** | **KHANA** | WPR | Urban | $316,420 | 839 | $377 |
| **Average cost ratio** | | | | | | $211 |
| **Pregnant women/pediatric TB cases** | | | | | | |
| **W2** | **KHANA** | WPR | Urban | $316,420 | 839 | $377 |
| **A1** | **HEAAI** | AFR | Urban | $491,441 | 494 | $995 |
| **A4** | **SHDEP** | AFR | Urban (General population; Children, Female Sex Workers, Small-Scale Miners, MSM) | $295,736 | 122 | $2,424 |
| **A11** | **NAANK** | AFR | Rural | $51,528 | 15 | $3,435 |
| **Average cost ratio** | | | | | | $786 |
| **Door-door screening** | | | | | | |
| **S5** | **INNOV** | SEAR | Rural | $308,777 | 1,099 | $281 |
| **A4** | **SHDEP** | AFR | Urban (General population; Children, Female Sex Workers, Small-Scale Miners, MSM) | $295,736 | 122 | $2,424 |
| **Average cost ratio** | | | | | | $495 |
| **Average cost ratio (All Projects)** | | | | | | $451 |

a. Region is grouped by the WHO definition: African Region (AFR), Region of the Americas (PAR), South-East Asia Region (SEAR), European Region (EUR), Eastern Mediterranean Region (EMR), and Western Pacific Region (WPR).

b. Projects are categorized into urban or rural setting based on the primary implementation environments. Targeted population is specified when being emphasized in the TB REACH narrative reports.

c. Cost per treatment completed is calculated as total project costs divided by a number of patients completed treatment.

**Table S9. Sensitivity analysis assessing the effects of changes in total cost and number of people diagnosed on the cost-effectiveness ratio per case diagnosed in case-finding only projects.**

| **Case-finding only Project** | | **Total Cost +/- 25%^a^** | | | **Number Diagnosed +/- 25%^b^** | | |
| --- | --- | --- | --- | --- | --- | --- | --- |
| **#** | **Code** | **Base Value** | **Low Value** | **High Value** | **Base Value** | **Low Value** | **High Value** |
| **S3** | **TBALI** | $30 | $22 | $37 | $30 | $24 | $39 |
| **S1** | **ICDDR** | $46 | $34 | $57 | $46 | $37 | $61 |
| **S2** | **REACH** | $108 | $81 | $135 | $108 | $86 | $144 |
| **E1** | **MERCY** | $231 | $173 | $289 | $231 | $185 | $308 |
| **A2** | **GOMSA** | $236 | $177 | $295 | $236 | $189 | $314 |
| **E2** | **ACREO** | $459 | $344 | $573 | $459 | $367 | $611 |
| **S8** | **MAPIN** | $589 | $441 | $736 | $589 | $471 | $785 |
| **A8** | **LSTMN** | $691 | $518 | $863 | $691 | $553 | $921 |
| **A6** | **CHEAS** | $1,937 | $1,453 | $2,422 | $1,937 | $1,550 | $2,583 |
| **A9** | **FUNDA** | $3,094 | $2,321 | $3,868 | $3,094 | $2,475 | $4,126 |
| **A10** | **IRDSA** | $10,497 | $7,873 | $13,122 | $10,497 | $8,398 | $13,996 |

a. Low and high values represent 25% adjustment in total costs reported by each project, where lower costs correspond to low ratio value.

b. Low and high values represent 25% adjustment in total number of case detection reported by each project, where lower costs correspond to high ratio value.

**Table S10. Sensitivity analysis assessing the effects of changes in cost and effectiveness on the cost per case diagnosed in projects with multiple objectives.**

| **Project with treatment** | | **Total Cost +/- 25%^a^** | | | **Number Diagnosed +/- 25%^b^** | | | **Number Treatment Initiated +/- 25%^c^** | | | **Number Treatment Completed +/- 25%^d^** | | |
| --- | --- | --- | --- | --- | --- | --- | --- | --- | --- | --- | --- | --- | --- |
| **#** | **Code** | **Base Value** | **Low Value** | **High Value** | **Base Value** | **Low Value** | **High Value** | **Base Value** | **Low Value** | **High Value** | **Base Value** | **Low Value** | **High Value** |
| **S4** | **ASHAK** | $103 | $77 | $128 | $103 | $79 | $143 | $123 | $98 | $163 | - | - | - |
| **W1** | **CATAC** | $141 | $105 | $176 | $141 | $111 | $190 | $155 | $124 | $207 | $8 | $6 | $11 |
| **W2** | **KHANA** | $152 | $114 | $190 | $152 | $115 | $214 | $196 | $157 | $261 | $84 | $68 | $113 |
| **S5** | **INNOV** | $160 | $120 | $200 | $160 | $125 | $219 | $188 | $151 | $251 | $29 | $23 | $39 |
| **A1** | **HEAAI** | $272 | $204 | $340 | $272 | $209 | $378 | $326 | $261 | $435 | $160 | $128 | $213 |
| **A5** | **LSTME** | $280 | $210 | $350 | $280 | $217 | $385 | $321 | $257 | $428 | - | - | - |
| **A4** | **SHDEP** | $303 | $227 | $378 | $303 | $239 | $409 | $343 | $274 | $457 | $137 | $109 | $182 |
| **S9** | **RUMAH** | $311 | $234 | $389 | $311 | $242 | $428 | $362 | $290 | $483 | - | - | - |
| **E3** | **BRICF** | $356 | $267 | $444 | $356 | $280 | $482 | $387 | $310 | $516 | $28 | $23 | $38 |
| **S7** | **OPASH** | $415 | $311 | $518 | $415 | $336 | $571 | $497 | $397 | $662 | - | - | - |
| **A3** | **CIDRZ** | $420 | $315 | $525 | $420 | $325 | $579 | $486 | $389 | $648 | - | - | - |
| **S6** | **BNMTN** | $424 | $318 | $530 | $424 | $319 | $577 | $453 | $363 | $604 | $28 | $23 | $38 |
| **A7** | **GLRAN** | $438 | $328 | $547 | $438 | $344 | $595 | $495 | $396 | $660 | - | - | - |
| **W3** | **VNTPV** | $511 | $383 | $639 | $511 | $407 | $684 | $638 | $510 | $851 | - | - | - |
| **W4** | **FITVT** | $739 | $554 | $924 | $739 | $587 | $992 | $789 | $631 | $1,052 | - | - | - |
| **A11** | **NAANK** | $2,056 | $1,542 | $2,570 | $2,056 | $1,627 | $2,770 | $2,147 | $1,718 | $2,863 | $146 | $117 | $195 |
| **P1** | **ASOCI** | $3,233 | $2,425 | $4,041 | $3,233 | $2,576 | $4,329 | $4,294 | $3,435 | $5,725 | $92 | $73 | $122 |
| **A12** | **GLOHI** | $9,913 | $7,435 | $12,391 | $9,913 | $7,803 | $13,437 | $10,608 | $8,486 | $14,144 | - | - | - |

a. Low and high values represent 25% adjustment in total costs reported by each project, where lower costs correspond to low ratio value.

b. Low and high values represent 25% adjustment in total number of case detection reported by each project, where lower costs correspond to high ratio value.

c. Low and high values represent 25% adjustment in total number of case initiated treatment by each project, where lower costs correspond to high ratio value.

d. Low and high values represent 25% adjustment in total number of case completing treatment by each project, where lower costs correspond to high ratio value.

**Table S11 Data Abstraction Tool with example**

| **Program Information** | | | | | **Costs** | | | | | | |
| --- | --- | --- | --- | --- | --- | --- | --- | --- | --- | --- | --- |
| **Project Title** | **#** | **PROJECT CODE** | **Country** | **Region Grouping** | **"Human Resources"** | **"Activities"** | **"Operational Research"** | **"Procurement of Medical Items"** | **"Procurement of Non-Medical Items"** | **"Direct Program Support"** | **Cumulative Expenditure** |
| Health Alliance International | A2 | HEAAI | Mozambique | African Region | $138,277 | $197,862 | $36,537 | $20,760 | $52,981 | $81,561 | $491,441 |
| **Presumptive TB** | **Diagnosis** | | | **Initiation** | | | **Treatment** | | | **Additional Notes** | |
| **Achieved number of presumptive TB** | **Cost-Diagnosed** | **Number diagnosed with TB (all forms)** | **Ratio-Diagnosed** | **Cost-Initiation** | **Number started on treatment** | **Ratio-Initiation** | **Cost-Treatment** | **Number of all forms TB patients successfully treated** | **Ratio-Treatment** | **Notes/project issues/possible reason for extreme values** | **Data quality issues** |
| 7921 | 412494 | 1516 | 272 | 491441 | 1507 | 326 | 491441 | 494 | 995.0000 | Study scaled up GxAlert application for use with GeneXpert results, created a OpenLDR database, and instituted a new DR-TB ECHO Videoconferencing and Telementoring Platform. | *NA* |

**
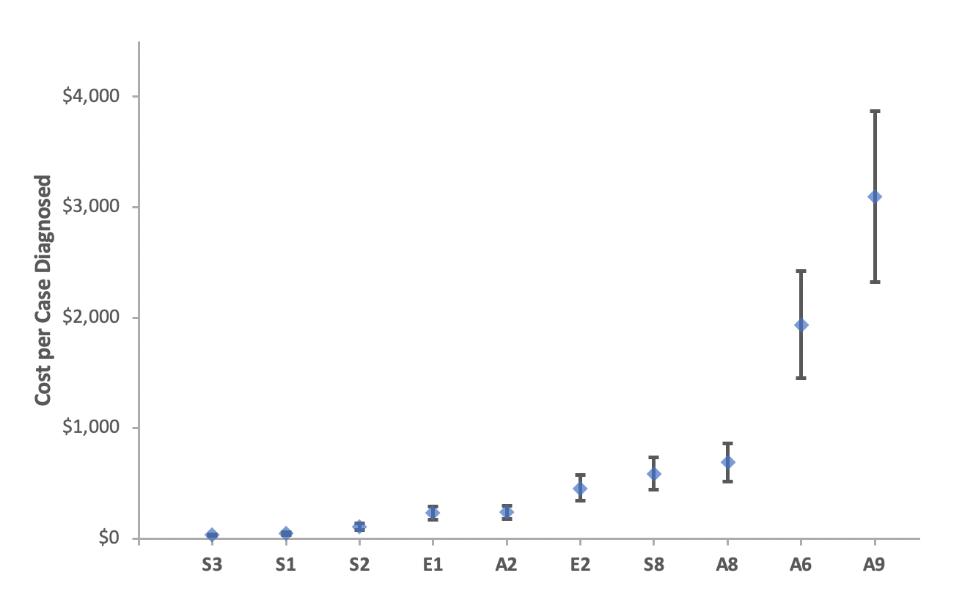
**

**Fig S1. Sensitivity Analysis on the Cost Effectiveness Ratio per Case Diagnosed with Total Cost +/- 25% for case-finding only projects.** For each case-finding only project, the blue diamond reflects the base value of the cost-effectiveness (CE) ratio, and the line represents the range of CE ratios when total costs fluctuate +/- 25% (from the upper bar to the bottom bar). Letters of the codes on the x-axis represent the geographic region in which the projects were performed, and numbers order projects from largest (1) to smallest within each region (e.g. S1 represents the project with the largest size in SEAR). The y-axis describes the scope and direction of the effect of varying total costs on the CE ratio of each project. The upper bar corresponds to the higher cost in each range (+25%), whereas the lower bar corresponds to the lower cost (-25%). Cost per case diagnosed is calculated as respective case-finding costs divided by the number of patients diagnosed. Projects FUNDA, IRDSA were removed from the figure due to extremeness.

**
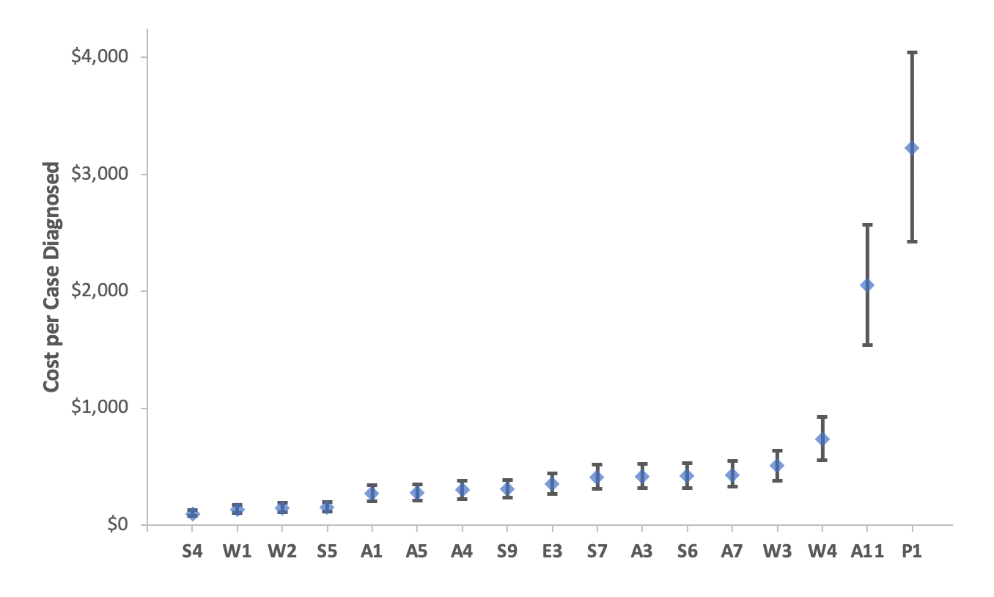
**

**Fig S2. Sensitivity Analysis on the Cost Effectiveness Ratio per Case Diagnosed with Total Cost +/- 25% for projects with case-finding and treatment.** For each dual-purpose project, the blue diamond reflects the base value of the CE ratio, and line represents the range of ratio when total costs fluctuates +/- 25% (from the upper bar to the bottom bar). Letters of the codes on x-axis represent the geographic region in which the projects were performed, and numbers order projects from largest (1) to smallest within each region (e.g. A1 represents the project with the largest size in AFR). The y-axis describes the scope and direction of the effect of the total costs change on the CE ratio of each projects. The upper bar corresponds to the higher cost in each range (+25%), whereas the lower bar corresponds to the lower cost (-25%). Cost per case diagnosed is calculated as case-finding costs divided by the number of patients diagnosed. Projects NAANK, ASOCI, GLOHI were removed from the figure due to extremeness.

**
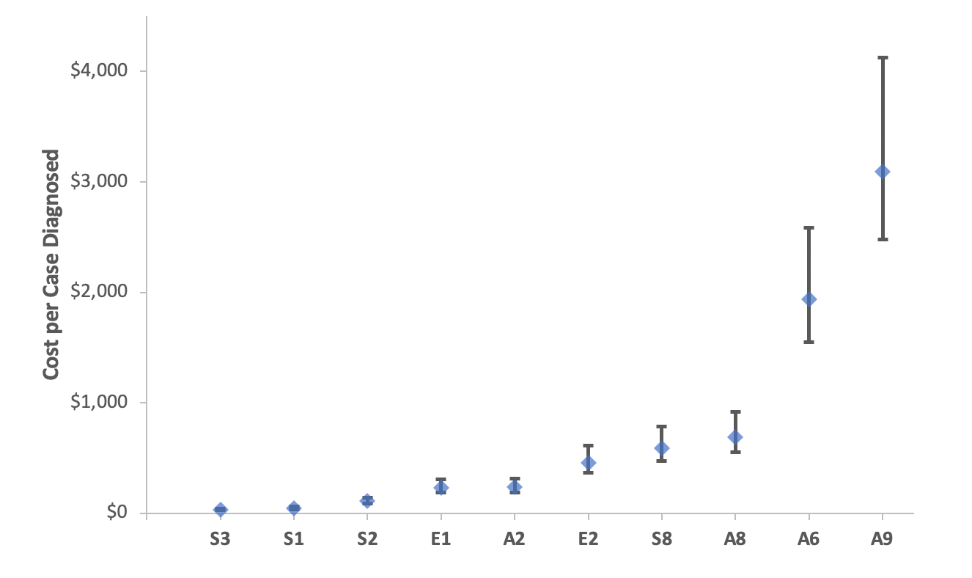
**

**Fig S3. Sensitivity Analysis on the Cost Effectiveness Ratio per Case Diagnosed with Number Diagnosed +/- 25% for case-finding only projects.** For each case-finding only project, the blue diamond reflects the base value of the CE ratio, and line represents the range of ratio when number of patients diagnosed fluctuates +/- 25% (from the bottom bar to the upper bar). Letters of the codes on x-axis represent the geographic region in which the projects were performed, and numbers order projects from largest (1) to smallest within each region (e.g. S1 represents the project with the largest size in SEAR). The y-axis describes the scope and direction of the effect of the number of patients diagnosed change on the CE ratio of each projects. The upper bar corresponds to the higher cost in each range (+25%), whereas the lower bar corresponds to the lower cost (-25%). Cost per case diagnosed is calculated as respective case-finding costs divided by a number of patients diagnosed. Projects FUNDA, IRDSA were removed from the figure due to extremeness.

**
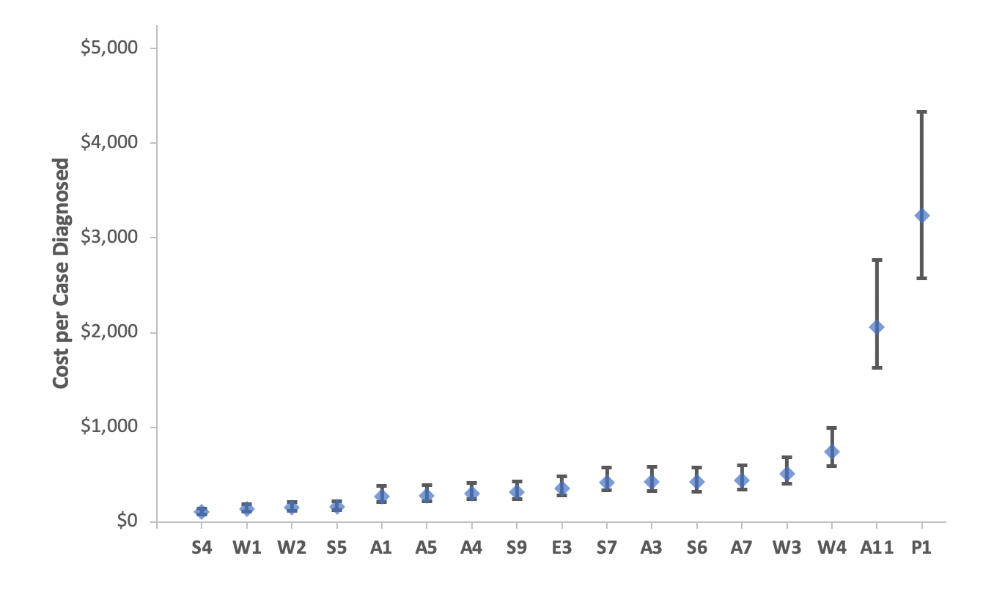
**

**Fig S4. Sensitivity Analysis on the Cost Effectiveness Ratio per Case Diagnosed with Number Diagnosed +/- 25% for projects with case-finding and treatment.** For each dual-purpose project, the blue diamond reflects the base value of the CE ratio, and line represents the range of ratio when number of patients diagnosed fluctuates +/- 25% (from the bottom bar to the upper bar). Letters of the codes on x-axis represent the geographic region in which the projects were performed, and numbers order projects from largest (1) to smallest within each region (e.g. A1 represents the project with the largest size in AFR). The y-axis describes the scope and direction of the effect of the number of patients diagnosed change on the CE ratio of each projects. The upper bar corresponds to the higher cost in each range (+25%), whereas the lower bar corresponds to the lower cost (-25%). Cost per case diagnosed is calculated as respective case-finding costs divided by a number of patients diagnosed. Projects NAANK, ASOCI, GLOHI were removed from the figure due to extremeness.

**
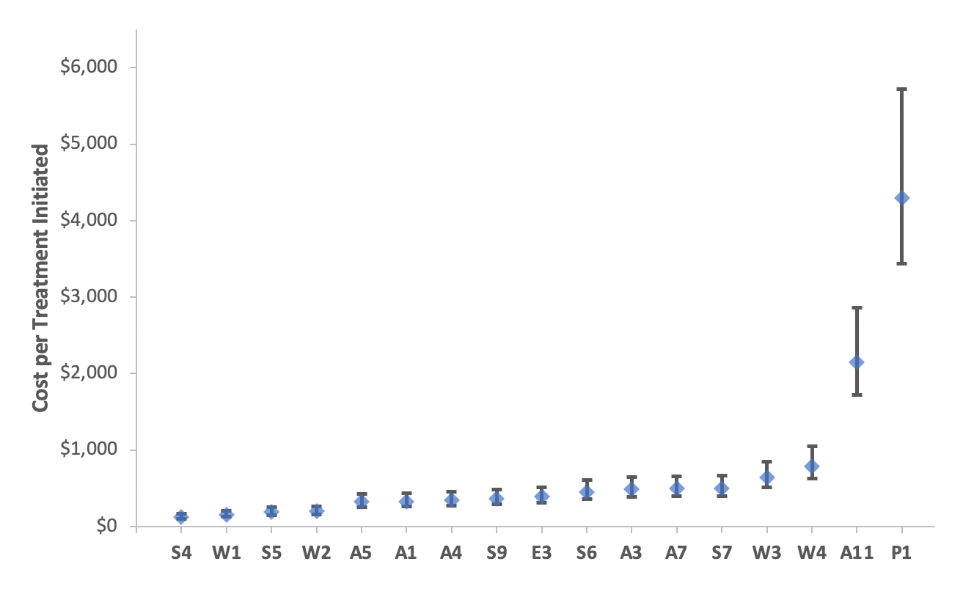
**

**Fig S5. Sensitivity Analysis on the Cost Effectiveness Ratio per Treatment Initiated with Number Initiated +/- 25% for projects with case-finding and treatment.** For each dual-purpose project, the blue diamond reflects the base value of the CE ratio, and line represents the range of ratio when number of patients initiated on treatment fluctuates +/- 25% (from the bottom bar to the upper bar). Letters of the codes on x-axis represent the geographic region in which the projects were performed, and numbers order projects from largest (1) to smallest within each region (e.g. A1 represents the project with the largest size in AFR). The y-axis describes the scope and direction of the effect of the number of patients change on the CE ratio of each projects. The upper bar corresponds to the higher cost in each range (+25%), whereas the lower bar corresponds to the lower cost (-25%). Cost per treatment initiation is calculated as total project costs divided by a number of patients initiated on treatment. Projects NAANK, ASOCI, GLOHI were removed from the figure due to extremeness.


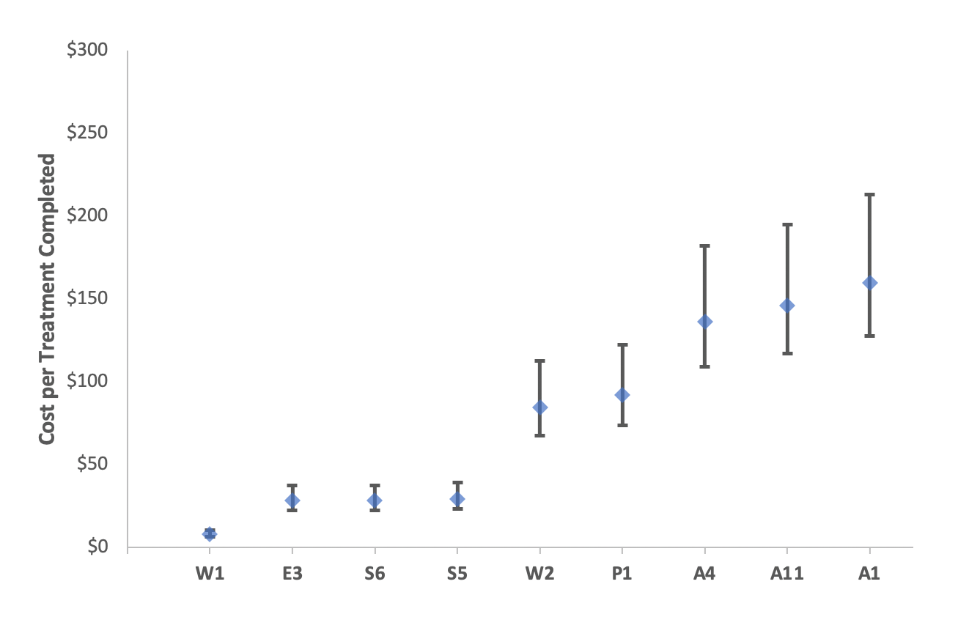


**Fig S6. Sensitivity Analysis on the Cost Effectiveness Ratio per Treatment Completed with Number Completed +/- 25% for projects with case-finding and treatment.** For each dual-purpose project, the blue diamond reflects the base value of the CE ratio, and line represents the range of ratio when number of patients completed treatment fluctuates +/- 25% (from the bottom bar to the upper bar). Letters of the codes on x-axis represent the geographic region in which the projects were performed, and numbers order projects from largest (1) to smallest within each region (e.g. A1 represents the project with the largest size in AFR). The y-axis describes the scope and direction of the effect of the number of patients change on the CE ratio of each projects. The upper bar corresponds to the higher cost in each range (+25%), whereas the lower bar corresponds to the lower cost (-25%). Cost per treatment completed is calculated as total project costs divided by a number of patients completed treatment. Projects NAANK, ASOCI, GLOHI were removed from the figure due to extremeness.
