# Supplementary material for: Comparative assessment of the cost-effectiveness of Tuberculosis (TB) active case-finding interventions: A systematic analysis of TB REACH wave 5 projects
Source: PLoS One. 2022 Sep 26;17(9):e0270816. doi: 10.1371/journal.pone.0270816 (PMC9512197; doi:10.1371/journal.pone.0270816)
Supplement: Attachment — Submitted filename: Response to Reviewers.docx [file pone.0270816.s002.docx]

May 23, 2022

Responses to the reviewer comments provided for PONE-D-21-13868R1

Manuscript Title: Comparative Assessment of the Cost-Effectiveness of Tuberculosis (TB) Active Case-Finding Interventions: A Systematic Analysis of TB REACH Wave 5 Projects.

Dear Editors and Editorial Team members,

We are grateful for the opportunity to revise and submit an improved manuscript, reflecting valuable feedback from the editors and reviewers. Specific responses to the editorial and reviewers’ comments are provided in blue, with references (with line numbers) to specific revisions made in the main manuscript (provided with italicized text with double quotes). A track change version of the revised manuscript and supplementing documents are uploaded with ‘R2’ designation.

Also, please note that the corresponding author’s (Hojoon Sohn) affiliation has chanced since the original submission. Details of this change have also been reflected in the revised version of this manuscript.

We believe that the editor and reviewers’ feedback have resulted in an improved manuscript and hope the editorial team and the reviewers agree.

Thank you.

Sincerely,

Hojoon Sohn, PhD MPH

Department of Preventive Medicine

Seoul National University

College of Medicine

Room #503, 103 Daehak-ro, Jongno-Gu

Seoul, Republic of Korea (03080)

+82-10-4707-5734

Reviewer #3: This is a revision of a previously submitted manuscript by Gomes and colleagues detailing a comparative assessment of different case finding approaches employed in TB REACH Wave 5 grants.

Comments

-Abstract, Purpose: Replace the words “TB control” with “TB prevention and care” in line with guidance about reducing the use of potentially stigmatizing language in TB research.

Response to Comment #1: Thank you for noting this important issue. We have reviewed our manuscript and replaced any potentially stigmatizing language in TB (e.g. replaced the phrase “TB control” with “TB prevention and care.”)

-Abstract, Methods/Results: How was the following calculated: “…with the corresponding country’s per-capita GDP ($543 per $1000 increase, 95% confidence interval: -$53, $1138).” I see no mention of this in the main text. Please remove or report in the main text, as well as the methods used to estimate this. Apologies if I missed this.

Response to Comment #2: We appreciate the Reviewer pointing this out and have added this result in the main text as well.

-Introduction, line 59: Please update with 2020 data and reference TB as the second leading cause of death due to an infectious disease (trailing SARS-CoV-2).

Response to Comment #3: Thank you for this comment. We have updated with 2021 data and reference TB and revised the text as the following:

*Line 60-62: “Tuberculosis (TB) is the second leading infectious cause of morbidity and mortality worldwide, trailing after SARS-CoV-2, with an estimated 9.9 million new TB cases and 1.5 million deaths in 2020.”*

-Line 68: I believe the same data is estimated annually in the Global TB Report (case under-ascertainment). It would be useful to reference the most recent data, in light of the ongoing pandemic.

Response to Comment #4: Thank you for this suggestion. We have updated the text in line 70-72 to reflect the most recent data estimated in the 2021 Global TB report:

*“Currently, it is estimated that about 30% of people who develop active TB every year will not be notified to public health authorities – largely reflecting underdiagnosis and undertreatment.”*

-Introduction: Did any TB REACH project employ “intensified case finding” – such as adding TB screening to routine clinic visits? It would be good to mention this as another method of case detection. I think the authors have grouped ICF under the ACF umbrella – which is fine, but there is a bit of nuance as ICF is generally less resource intensive.

Response to Comment #5: We have modified the introduction and Table 2 to include mention of intensified case finding, as 12 TB REACH projects employed this intervention.

-Methods, line 167: Is there a figure supplied which details eligible projects and reasons for exclusion?

Response to Comment #6: Thank you for this question. We did not develop a diagram to illustrate inclusion/exclusion of the projects in our study as our original aim was to evaluate all funded projects. We excluded a total of three projects (NTRL, EPHI; Ifakara; and AIGHD) upon detailed review on the basis that they did not report relevant cost or program yield/performance data to TB REACH. The decision to exclude these projects were made together with the TB REACH technical officers, as described in lines 124-133:

*“Wave 5 projects that did not report relevant cost or program yield/performance data to TB REACH were excluded from the study. After consultation with TB REACH technical officers, three additional projects were excluded from the analysis. The first project (NTRL, EPHI) was a lab-based assessment of a novel transport and decontaminating reagent for TB testing, called OMNIgene® SPUTUM. The second project (Ifakara Health Institute) was intended to assess the use of Xpert Omni/Xpert MTB Ultra cartridges. However, these cartridges were unavailable during the wave 5 funding cycle; therefore, the project was not able to begin activities. The third project (AIGHD) aimed to establish TB screening in an HIV community testing project that was similarly postponed.”*

Methods, Cost Apportioning: I am curious how the authors dealt with capital costs in projects? Having reviewed some TB REACH expenditures myself, this is problematic. For example, software is purchased or a diagnostic modality is purchased, however the included participants are only a subset of the patients that would be eligible outside of an operational research project. If the diagnostic modality is expected to last beyond the project, including its full cost would overestimate costs. Further, if only a subset of patients are included, then CER may be overestimated as in practice, more patients would use the intervention, reducing the ‘cost per patient’ for fixed costs. I don’t see a mention of how this was handled in the methods and I think it needs to be addressed somewhere.

Methods, Human Resource Costs: Can the authors comment on how human resources were considered? Would routine personnel implementing an intervention as part of a TB REACH project be included in reports? For example, if a healthcare worker at an antenatal clinic is now tasked with TB symptom screening as part of the study, would their additional time/cost appear in the human resources cost? If not or if this is uncertain, it must be listed as a limitation.

Response to Comments #7 and #8: Thank you very much for these insightful comments. We agree with the reviewer comments and to follow the reviewer’s suggestion that a detailed review of itemized finance and activity reports is needed. Also, to allow comparative assessment across the projects, reporting of these cost data must be standardized. Unfortunately, the data available to us did not allow for comparison of detailed cost data across the projects. Most projects reported limited or incomplete information on the cost data; therefore, we were not able to properly apportion or annuitize costs to relevant sub-activities of ACF. Furthermore, no projects reported costs or human resource efforts that were outside of the direct programmatic costs and efforts (i.e. there was no information that we could ascertain to assess human resource costs that were not compensated through the project budget). As the reviewer suggested, these limitations in data may result in our cost and cost-effectiveness estimates being either under or overestimated (depending on the sources and types of these data limitations). We have therefore added text in our discussion section to clarify these limitations and how our results should be interpreted in light of these limitations in lines 399-421:

*“**There are several limitations to consider when interpreting our study’s findings. First, cost and project activity data available (level of data) for each project were highly heterogenous in that systematic assessment of activity-based costs and declassification cost data (e.g. capital assets, fixed and variable costs) across the projects were not possible. As such, we were only able to use simplified apportionment criteria to allocate total costs for major programmatic activities only: case finding, treatment initiation (linkage to care), and treatment management. This may have resulted in over-estimation of costs as certain capital assets and fixed cost items may have value beyond project activity (e.g. laboratory equipment).* *Additionally, several Wave 5 projects reported that provision of (e.g., to contacts of TB patients identified during ACF campaign) TB preventive therapy (TPT). However, we were unable to categorically assess costs nor performance outcomes explicitly for activities relating to TPT from data sources available to our team, including project’s financial statements. Therefore, misclassification of TPT related costs, while it may be small, may have resulted in over-estimation of CERs for these projects. We also were not able to extract quantifiable data on human resource involvement that are were not compensated through the project budget (e.g. routine healthcare worker tasked to screen TB symptoms at antenatal clinic for ICF). In this regard, our results may be an under-estimate of real-world human resource costs of ACF interventions. These data limitations in our study shed light on the need to improve and standardize the types and depth of the data necessary to perform comparative in-depth empiric cost and cost-effectiveness assessment of ACF interventions.”*

- Methods/Results, Efficiency of Programs: Did the authors further consider the efficiency of interventions? This is complementary to the CER. For example, it allows you to assess how many participants had to be screened to detect one case of TB and if efficiency impacted CER. If such data were not available in final reports, it should be noted. Relatedly and while outside the scope of your analysis, it would also be good to mention in discussion that cost per person screened is extremely useful to programs for budgeting purposes and is an area of further research.

Response to Comment #9: Thank you for this suggestion. We agree that the number needed to screen (NNS) is very useful estimate of efficiency and have now reported the NNS for all applicable projects in Table 3. We have also included the following in the Discussion (lines 421-423): “In particular, the cost per person screened (or initiating treatment) is also useful to programs for budgeting purposes and should be a priority for future research.”

Reviewer #4: This study presents a comparative analysis on cost-effectiveness of TB REACH Wave 5 projects, considering single measures of health outcome in the assessment. The analysis starts with a systematic review of projects, where the authors categorize the projects in the data extraction step. Then, they evaluate the projects by comparing their cost-effectiveness on single measures of health outcome: number people diagnosed, number of treatment initiations, number of treatment completions. The interventions used for active case finding (ACF) activities and the impact of these ACF interventions might be different in each project. So, it would be good to see a comparison between cost-effectiveness of active-case finding activities and passive, facility-based case-finding alone, since it is not intuitively clear what is cost-effective in the analyses. The cost-effectiveness ratio alone is not sufficient to determine if the benefit of an intervention is worth its cost. We would be able to say an intervention is cost-effective based on the comparison to another use of resources or some recognized standard.

Response to Comment #10: Thank you for your insightful comment. We agree with the reviewer that ‘cost-effectiveness’ of ACF interventions would be best assessed by estimating the incremental cost-effectiveness ratio (ICER), evaluated against ‘passive case-finding’ as a comparator. However, the diversity of the ‘baseline’ conditions for each project (e.g., differences in routine TB care practices and settings), target populations, and the intervention contents would require developing individual analyses for each project. In response to this comment, we have clarified our study’s objectives and rationale, as well as limitations that need to be considered (and improved in future research) in interpreting our findings. These described in following sections of our manuscript:

*Lines 90-100: “**Given the substantial investment made in these projects (over 155 million USD since the inception of the initiative), it is critical to understand the relative value generated by different types of case finding interventions. In this study, we used the project database of the TB REACH Wave 5 funded projects to comprehensively and systematically evaluate costs and cost-effectiveness across the wide range of case finding and treatment support projects supported by this initiative. In doing so, we compared cost-effectiveness ratios (CERs) [6] assessed for common outcome units that are most direct and readily calculated to measure ACF performance based on each project’s scope of operations (1. TB case finding; 2. Linkage to care; and 3. TB treatment support) to rank and compare various factors influencing CERs.”*

*Lines 425-438: “**Secondly, we did not use estimates of health utility such as QALYs and DALYs, as conversion from cases detected to these measures is inherently context-specific. Therefore, our study findings may not be comparable to projects with other health outcomes. However, the cost per TB case detected is arguably the most direct and readily calculated measure of ACF performance without the need to make additional strong assumptions (e.g., future trajectory of people with TB who are not detected by ACF, impact on transmission of delayed diagnosis). Earlier analyses that do make these assumptions suggest that, in high-burden countries where missed TB diagnosis is common, the cost per case detected is about 25% higher than the cost per DALY averted (when considered over a 10-year time horizon) [6]. Thus, our cost-effectiveness ratios in terms of cost per case detected are likely to be modestly higher than the corresponding cost per DALY averted – but still well within most country-specific cost-effectiveness thresholds [12].”*

*Lines 440-450: “**Thirdly, a simplified, standardized analytic approach taken in this study can be helpful for high-level comparisons on factors influencing cost-effectiveness (or costs associated programmatic performance) of interventions with similar/same objective. However, as described in our study, ACF projects are becoming more diversified to address gaps in the complex TB care cascade that are specific to the needs in the settings where these projects are implemented. Therefore, more detailed evaluation that includes projection of future benefits of key programmatic components (e.g. TPT provision to contacts of TB patients identified through ACF interventions or treatment linkages and adherence programs) is needed for each individual projects to precisely estimate the overall value and incremental cost-effectiveness of ACF interventions in each context [6, 7, 18-21].”*

Abstract: In the “Purpose” section, the main objective of the study needs to be described clearly.

Response to Comment #11: Thank you for this suggestion. We have clarified the main objective of the study in the Purpose section of the abstract:

*Lines 25-28: “In this study, we present a comparative analysis on cost-effectiveness of TB REACH Wave 5 projects with diverse programmatic objectives to inform future decisions regarding funding, strategic adoption, and scale-up.”*

- Introduction: The authors mentioned about the TB REACH initiative in general, but there is no information on why Wave 5 projects was chosen for the study. Was there a specific reason for analyzing the TB Reach Wave 5 projects or does this study serve as a representative / pilot study for the comparative assessment of the cost-effectiveness of TB REACH projects? The authors might add a short sentence to clarify it in the “Introduction” section. (In “Methods”, the sentence (lines 97-106) states that “the main focus of the funding cycle was TB case detection, the overall scope of projects funded was broad”. Was that the reason of considering Wave 5 projects in this study?) The last paragraph could be improved by adding a few sentences on the overall aim of the work and commenting whether that aim was achieved.

Response to Comment #12: We thank the reviewer for this comment. The reason for considering Wave 5 projects in this study was largely a pragmatic one, as this project was performed in conjunction with a specific review of Wave 5 projects in particular. In response to this comment, we have carefully reviewed the manuscript to ensure that all mentions of “TB Reach” in general versus “TB Reach Wave 5” are appropriate. We have also added the following sentence to the paragraph in the Introduction pointed out by the Reviewer (line 84-85): “In particular, Wave 5 – the focus of this analysis – focused on innovative approaches to case finding.”

With respect to the overall aim of the work, we focus on this in the first paragraph of the Discussion (Lines 331-332): “These findings demonstrate the importance of considering local context and realities of implementation when evaluating cost-effectiveness and argue against making blanket statements about the cost-effectiveness of certain interventions (e.g., TB case detection).

Furthermore, our specific results can be helpful to implementers and funders seeking to introduce interventions to strengthen the TB cascade of care in cost-effective fashion across a variety of diverse settings.”

Methods: As it can be seen from the Stop TB website ([https://stoptb.org/global/awards/tbreach/wave5.asp](https://nam02.safelinks.protection.outlook.com/?url=https%3A%2F%2Fstoptb.org%2Fglobal%2Fawards%2Ftbreach%2Fwave5.asp&data=04%7C01%7Chsohn6%40jhu.edu%7C84e081cf9fb84d1579c208d9b4d28bb1%7C9fa4f438b1e6473b803f86f8aedf0dec%7C0%7C0%7C637739638637364278%7CUnknown%7CTWFpbGZsb3d8eyJWIjoiMC4wLjAwMDAiLCJQIjoiV2luMzIiLCJBTiI6Ik1haWwiLCJXVCI6Mn0%3D%7C3000&sdata=geci%2B8FA2hotNjdPjCazsEVXyK2Wbc6xWSSSwiq%2FhC4%3D&reserved=0)), it was stated that there are 38 projects funded in Wave 5. But the paper says the number is 32. Why is there a difference? Also, in the paper, it was indicated that three projects were excluded from the analysis (lines 167-169). Is it excluding 3 projects from the 32 projects or the 29 projects? This could create confusion for the audience. My understanding is that the authors excluded 3 projects from the 32 projects, since all tables include 29 projects. It would be better to mention this (excluding 3 projects) before Table 1.

Response to Comment #13: Thank you for the comment. 38 projects were funded in Wave 5 but six of these projects were supported by small grant and focused on product development. The remaining 32 were case-finding projects and are the main focus of this study. Among the 32 projects, 3 were described and removed from the analysis. We have moved the text regarding exclusion of the three projects to earlier in the manuscript (to before Table 1, as suggested).

Table 1 (Project characteristics and description): If regions are abbreviated as AFR, PAR, SEAR, EUR, EMR, WPR, why were they written differently in this table? (all of them are ending with O)

Response to Comment #14: Thank you for this comment. We have updated the manuscript and supporting information, changing all abbreviations to AFR, PAR, SEAR, EUR, EMR, and WPR, for consistency and in accordance with their WHO designations.

Table 2 (Projects organized by subgroup): Letter subscript a was used for “Provision of preventive therapy”, but the corresponding footnote was not given at the end of the table.

Response to Comment #15: Thank you for noting this issue. The letter subscript “a.” was added to the corresponding footnote given at the end of the table.

Lines 115-123: What are the inclusion and exclusion criteria that two authors considered to perform the data extraction? It would be good to include them to clarify how this step was performed.

Response to Comment #16: Thank you for your question on this matter. First, we did not have specific *a priori* inclusion and exclusion criteria for selecting wave 5 projects as our original aim was to evaluate the cost-effectiveness for all projects. However, upon detailed review of each project, we discovered that certain projects were not suitable to calculate cost-effectiveness ratios (CERs) for case finding, linkage to care, and treatment support. Therefore, we excluded projects (three) that did not have data on costs or program yield/performance (e.g. due to projects did not operate due to unavailability of GeneXpert Omni instrument or were an exploratory assessment of sample transport reagent). Details on these decisions are reported in lines 172-181:

*“Wave 5 projects that did not report relevant cost or program yield/performance data to TB REACH were excluded from the study. After consultation with TB REACH technical officers, three additional projects were excluded from the analysis. The first project (NTRL, EPHI) was a lab-based assessment of a novel transport and decontaminating reagent for TB testing, called OMNIgene® SPUTUM. The second project (Ifakara Health Institute) was intended to assess the use of Xpert Omni/Xpert MTB Ultra cartridges. However, these cartridges were unavailable during the wave 5 funding cycle; therefore, the project was not able to begin activities. The third project (AIGHD) aimed to establish TB screening in an HIV community testing project that was similarly postponed.”*

Second, our criteria and scope of project-specific data extraction are provided in lines 157-168 and 183-185:

*Lines 157-168: “**Variables directly collected from financial statements included the characteristics of each program, the country in which the project was executed, a brief description of the program’s primary activities (i.e. community-based screening, scale-up of previous concepts, testing of new sample transport or drug delivery systems, etc.); location of project operations (facility-based, door-to-door screening by community health workers, etc.); detail regarding the program’s target population (i.e. general population, gender-based or geographical subpopulations, etc.); screening, diagnosis and treatment services; and available technology (e.g. mobile-health tools for screening, mobile chest X-rays, Xpert MTB/RIF assay), as well as reported expenditures. For each project, all financial items reported, including total budget, income received, and cumulative expenditure, were extracted separately and reported in 2017 US dollars.”*

*Lines 183-185: “**Additionally, we extracted data on project service outputs, including number of people diagnosed with any type of TB, number of people started on TB treatment (notifications), and number of patients successfully treated (Tables 3, S1 and S3).”*

Lines 212-213: There is no Box 3 in the manuscript or in the supporting document. There is no Box 2, either.

Response to Comment #17: Thank you for noting this error in our text. We have fixed this issue within the manuscript and supporting document, by deleting mention of Boxes 2 and 3, and replacing them with Tables 3, S1-S4, which include information on each project’s scope and additional details, matched to each text’s contents.

Results: Line 244 – 245: For this part “the weighted average CER per TB case detected across all projects”, please add reference to Table S5.

Response to Comment #18: Thank you for this suggestion. In reflecting on this comment, we felt that the information presented in Table 3 would be appropriately address this issue and have revised the text accordingly.

Lines 252-255: Similarly, for these projects, please add reference to Table S2 and S3, respectively.

Response to Comment #19: We have added the reference to Table S2 and S3, respectively.

Discussion: Lines 332-335: When comparing different types of projects, and considering some of them as “more cost-effective”, did the authors also look into the “less cost-effective” projects whether they experienced problems during implementing ACF activities? Were the “four projects” (lines 359-375) excluded from the analyses when commenting based on the comparison of the cost-effectiveness ratios (lines 332-335)?

Response to Comment #20: Thank you for this comment. First, we have provided the details on four projects that had highest cost-effectiveness ratios (CERs) in lines 381-391. As the reviewer may see in the text below, these projects generally confronted operational and implementation challenges (e.g. delays in hiring and retaining staff, political unrest, security concerns for household visits, and technical failures of novel technologies such as drones):

*“Four projects were identified as having higher cost-effectiveness ratios. On deeper review, these projects each confronted major operational difficulties during implementation. CHEAS experienced a major delay in implementation owing to challenges in hiring and retaining staff and underlying political unrest. IRDSA reported challenges in making household visits due to security concerns and a lower-than-expected number of patients with undiagnosed TB seeking services in the clinics.* *GLOHI reported that TB incidence in their region may have been overestimated (thereby resulting in fewer patients identified) and experienced multiple technical failures in drone usage, which hindered efficiency and implementation. The FUNDA project was substantially delayed in receipt of ethical approval, leading to lower-than-expected rates of TB diagnosis.”*

As projects did not document the operational, implementation, and technical challenges in a standardized fashion, it is difficult to systematically assess how these challenges have impacted CERs. Therefore, we’ve limited our interpretation on this matter as the following (lines 391-397).

*“These logistical challenges speak to the unpredictable nature of scaling up health interventions in real-world settings and the resulting variation in cost-effectiveness that will likely be observed in actual implementation. These project-specific findings should not be interpreted as favoring one intervention over another – as such barriers to implementation are generally unexpected and often not related to the actual type of intervention performed.”*

Second, we did not exclude these four projects that had highest cost-effectiveness ratios (CERs) when commenting on the overall CERs (weighted average cost per case detected) in lines 256-260.

Reviewer #5: While this is a relatively simple analysis, comparative assessments of costs and outcomes across projects is sorely needed and will be of great benefit to both researchers and funding sources for guidance on prioritizing funding decisions across projects. I commend the authors for this work. My comments reflect changes that will make the paper more cohesive and highlight outcomes that would be of more interest to the reader.

General comments: In general, the paper goes back and forth between different phrases, which makes it a bit difficult to read. This includes using two different codes for each project, switching between ACF and TB case-finding, and treatment initiation vs. linkage to care. Since the paper is reviewing multiple projects across many contexts, which can be confusing for the reader, the authors should ensure that the rest of the paper is as cohesive and streamlined as possible. There are also a few results that I believe should be highlighted and expounded upon.

Thank you for your general comments. We have reviewed and revised our manuscript to be more consistent in the language in describing the categorization of projects as 1) TB case finding; 2) linkage to TB care; and 3) TB treatment support. For other specific comments and issues raised, we’ve provided our responses below each comment.

Methods:

1) I would be more explicit about the costing methodology, as this sounds like macro/gross costing. Would it be possible to share a blank version of the data extraction spreadsheet in the appendix? This might help the reader understand exactly what data were extracted from the financial reports.

Response to Comment #21: Thank you for this suggestion. We added an abstraction tool as Table S11 with an example of project HEAAI.

2) Could the authors please specify the state of the projects in wave 5? Were all of the projects just starting in wave 5, or were some of them started in previous waves, and therefore only recurring costs were included in wave 5? This could potentially have an effect on CER, as start-up costs for some interventions can be quite high, and if not incorporated, can artificially lower CERs.

Response to Comment #22: Thank you for your suggestion. We have added the information regarding the state of the projects in the text and Table 1 using the TB REACH’s classification. We chose not to report the comparison of CERs considering the state of projects due to the small number of scaled-up projects by different project type.

3) Lines 155-159: Some projects involved the use of preventive therapy but costs for preventive therapy were not explicitly reported. Does this mean that you were not able to exclude the costs of preventive therapy from these projects, and therefore those costs are included in the CER of these projects? This needs to be explained more clearly (and if it is a limitation, discussed in limitations section).

Response to Comment #23: Thank you for this comment. Some Wave 5 projects have mentioned that project activities included provision of preventive therapy, but we were not able to categorically assess costs for activities relating to preventive therapy from all of our data sources, including project’s financial statements. Therefore, we were unable explicitly measure cost related to activities for identifying and providing patients (e.g. usually those who were contacts of TB patients identified during ACF campaign) in need of TB preventive therapy. We’ve revised the text in the discussion section to address these limitations in lines 408-414:

*“Additionally, several Wave 5 projects reported that provision of (e.g., to contacts of TB patients identified during ACF campaign) TB preventive therapy (TPT). However, we were unable to categorically assess costs nor performance outcomes explicitly for activities relating to TPT from data sources available to our team, including project’s financial statements. Therefore, misclassification of TPT related costs, while it may be small, may have resulted in over-estimation of CERs for these projects.”*

Results:
1) Lines 257 – 261: When referring to the projects with CER >$1000, it would be beneficial to mention which of these projects were case-finding only and which included linkage to care + patient support. --- Some of these programmatic setbacks are discussed in the Discussion section (lines 359-375), but it would still be a good idea to mention briefly in the Results section that these costs were inflated by programmatic setbacks and will be discussed further in the Discussion section.

Response to Comment #24: Thank you for your suggestion. For the projects with CER >$1000, we have added the text to specify type and describe programmatic setbacks in the result section (lines 270-277):

*“**Six projects were identified as projects with CERs above a $1,000-per-case-detected threshold. Two of these projects – NAANK and ASOCI – included treatment support efforts and were implemented in upper-middle income countries. Thus, only three case-finding only projects (CHEAS, IRDSA, and FUNDA) and one project with linkage-to-care (GLOHI) had an estimated cost per case diagnosed higher than the corresponding country’s per-capita GDP (Tables 1 and 3). Programmatic setbacks were the potential reasons for these inflated costs and will be discussed further in the Discussion section.”*

2) Lines 283 – 285, Figure 1. I am finding it confusing to refer to the projects by both their code names and also a separate alphanumeric code for the figures. Would it be possible to just use one code per project throughout the manuscript?

Response to Comment #25: Thank you for this comment. We feel that both ways of coding projects have their own advantages as the code names are representative of the project title while the alphanumeric code reflects the scale of the project and the region. To avoid confusion, we’ve added both code names in texts where applicable:

*Lines 300-304: “**In each panel, there was one project that was not shown because its associated cost-effectiveness ratio was exceptionally high (Panel A, project A10/IRDSA, cost per case diagnosed $10,497, GDP per capita: $6,133; Panel B, project A12/GLOHI, cost per case diagnosed $9,913, GDP per capita: $515).”*

3) Lines 287 – 294: These results may be important enough to include in the main figures rather than in the supplementary materials. Since there are a few projects with exceptionally high CER, it would be interesting to know what characteristics of those specific projects are driving those costs (if possible). I actually think this would be more important than the sensitivity analysis, which could be shortened/potentially moved to the supplementary materials.

Response to Comment #26: Thank you for this comment. We understand and agree with the reviewer that comparing CERs based on each project’s major programmatic components settings are important results. However, these data largely represent a restructured presentation of information in Tables 1-3 and do not – in our estimation – accurately depict the primary reasons that the specific projects noted had such high CERs. As noted by the Reviewer below, the fourth paragraph of the Discussion (lines 381-397) provides a detailed description of the four projects with the highest CERs – we feel that these descriptions provide a more accurate depiction of why the CERs were so high, rather than any specific data elements in this Table. Therefore, while we agree with the Reviewer that these data are important, we have retained this information in the supplementary document (Table S5).


Discussion:
1) Line 323: This seems to be the first time the “CET” acronym has been used in the text – please define this beforehand for the benefit of the layperson.

Response to Comment #27: Thank you for catching this error. We have adjusted the text to define the CET acronym when cost-effectiveness thresholds are first mentioned.

2) Line 332 and 359: Please remove the phrase in parentheses “(i.e., more cost-effective)”, as these are not ICERs and the CERs are not being compared to a common threshold. In line 359, I would also recommend removing the phrase in parentheses “(i.e,. less favorable)”, as it is a judgment call.

Response to Comment #28: Thank you for the detailed feedback. We have removed both phrases in parentheses from the Discussion section.

3) Line 365: I would include a little bit more discussion on how incidence of TB affects CER especially of case-finding interventions, as CERs will be much higher in a low incidence setting than high incidence. This may not be very obvious to the layperson, but could be very important in funding decisions. This commentary could be included around lines 335-338, where the authors mention that the cost of treatment support is lower than the cost of diagnosis, and then tied back in to the paragraph describing the four high CER projects in lines 360 -375.

Response to Comment #29: We agree with the Reviewer that the underlying incidence is a primary driver of the cost-effectiveness of active case finding. We have included the following sentence in the paragraph in question (now lines 358-360): “Cost-effectiveness is also more favorable in settings with higher TB incidence, as the costs of screening result in more people with TB detected and treated.” We then circle back with a parenthetical comment when describing the GLOHI project as one of the four projects with a high CER in lines 387-388: “GLOHI reported that TB incidence in their region may have been overestimated (thereby resulting in fewer patients identified).”
.
4) Line 380 – “reflected the available data that was available” is repetitive. Perhaps “paucity of available data…”?

Response to Comment #30: Thank you for this detailed assessment. We have replaced the repetitive phrase with “the paucity of available data” as suggested.

5) I know another reviewer suggested including DALYs in the analysis, but I don’t think it makes much sense with the purpose of this paper as the effectiveness outcomes are all based on programmatic outcomes, and not meant to be compared across interventions. I know another reviewer requested the consideration of the DALY, but I think discussion of the DALY takes up space in the Discussion section that could be better used to reflect on the cost drivers of the high CER interventions and the results of the subgroup analyses. I leave this to the authors’ discretion.

Response to Comment #31: Thank you for your comment. The reviewer is correct that calculation of DALY would be useful, but for comparing cost-effectiveness across same/similar types of intervention, we felt that evaluating cost-effectiveness based on most directly measurable outcome/performance was adequate. In this case, cost per case detected, patients linked to TB treatment, and TB treatment completed were common performance measure that could be applied to Wave 5 projects. As noted by the reviewer, we have included commentary on the limitations of our approach and how our findings should be interpreted in the Discussion section.
